# Supplementary material for: Deletion of wfs1 Impairs Oligodendrocyte Precursor Cells Dorsal Distribution and Myelination Through the wfs1-hmgcs1 Axis in Zebrafish
Source: Biology (Basel). 2026 Aug 16;15(16):1402. doi: 10.3390/biology15161402 (PMC13510151; doi:10.3390/biology15161402)
Supplement: Supplementary file 1 [file biology-15-01402-s001.zip › File S1-Transcriptome sequencing data and differentially expressed genes (DEGs) in wfs1-- zebrafish at 5 dpf/2.KEGG/wfs1KOvsWT/GSEA/wfs1KO/FATTY_ACID_METABOLISM/FATTY_ACID_METABOLISM(DRE01212).html]

Details for gene set FATTY\_ACID\_METABOLISM(DRE01212)[GSEA]

|  || Dataset | deg.sample |
| Phenotype | sample.cls |
| Upregulated in class | wfs1KO |
| GeneSet | FATTY\_ACID\_METABOLISM(DRE01212) |
| Enrichment Score (ES) | 0.34646624 |
| Normalized Enrichment Score (NES) | 1.5138196 |
| Nominal p-value | 0.036 |
| FDR q-value | 0.23665278 |
| FWER p-Value | 0.603 |
Table: GSEA Results Summary

  

Fig 1: Enrichment plot: FATTY\_ACID\_METABOLISM(DRE01212)      
 Profile of the Running ES Score & Positions of GeneSet Members on the Rank Ordered List

  

| PROBE | DESCRIPTION (from dataset) | GENE SYMBOL | GENE\_TITLE | RANK IN GENE LIST | RANK METRIC SCORE | RUNNING ES | CORE ENRICHMENT || 1 | 393441 | hacd3 |  |  | 1017 | 2.075 | 0.0068 | Yes |
| 2 | 140615 | fads2 |  |  | 1590 | 1.600 | 0.0187 | Yes |
| 3 | 553401 | hadhaa |  |  | 1608 | 1.593 | 0.0479 | Yes |
| 4 | 100005717 | cpt2 |  |  | 1666 | 1.560 | 0.0753 | Yes |
| 5 | 559403\_1 | acaca |  |  | 1982 | 1.374 | 0.0910 | Yes |
| 6 | 554145 | elovl8b |  |  | 2103 | 1.324 | 0.1120 | Yes |
| 7 | 334217\_1 | elovl7a |  |  | 2141 | 1.310 | 0.1353 | Yes |
| 8 | 393135 | zgc:55621 |  |  | 2338 | 1.233 | 0.1522 | Yes |
| 9 | 394156\_1 | acadl |  |  | 2548 | 1.160 | 0.1673 | Yes |
| 10 | 449816 | elovl1a |  |  | 2673 | 1.120 | 0.1843 | Yes |
| 11 | 327274 | elovl7b |  |  | 2805 | 1.083 | 0.2004 | Yes |
| 12 | 368367\_1 | hsd20b2 |  |  | 2911 | 1.057 | 0.2169 | Yes |
| 13 | 103909985 | LOC103909985 |  |  | 3098 | 1.013 | 0.2299 | Yes |
| 14 | 100037342 | acadsb |  |  | 3276 | 0.978 | 0.2426 | Yes |
| 15 | 393622 | acsl4a |  |  | 3304 | 0.972 | 0.2599 | Yes |
| 16 | 393657 | zgc:66024 |  |  | 3724 | 0.885 | 0.2633 | Yes |
| 17 | 445288 | acads |  |  | 3897 | 0.848 | 0.2737 | Yes |
| 18 | 678614 | elovl2 |  |  | 3951 | 0.839 | 0.2877 | Yes |
| 19 | 30643 | acat2 |  |  | 4174 | 0.798 | 0.2957 | Yes |
| 20 | 406830 | zgc:55413 |  |  | 4240 | 0.787 | 0.3083 | Yes |
| 21 | 100000859 | ehhadh |  |  | 4565 | 0.737 | 0.3119 | Yes |
| 22 | 335732 | elovl4b |  |  | 4729 | 0.714 | 0.3201 | Yes |
| 23 | 562292\_2 | acsf3 |  |  | 4884 | 0.696 | 0.3283 | Yes |
| 24 | 100006023 | acsbg1 |  |  | 5003 | 0.680 | 0.3373 | Yes |
| 25 | 559403 | acaca |  |  | 5108 | 0.666 | 0.3465 | Yes |
| 26 | 447860 | acsl5 |  |  | 5835 | 0.604 | 0.3349 | No |
| 27 | 449662 | acox1 |  |  | 6280 | 0.559 | 0.3314 | No |
| 28 | 336606 | hadhb |  |  | 6664 | 0.523 | 0.3291 | No |
| 29 | 553734 | scdb |  |  | 7381 | 0.461 | 0.3152 | No |
| 30 | 368367 | hsd20b2 |  |  | 8757 | 0.384 | 0.2792 | No |
| 31 | 402954 | cbr4 |  |  | 9038 | 0.365 | 0.2772 | No |
| 32 | 562292\_1 | acsf3 |  |  | 9095 | 0.360 | 0.2821 | No |
| 33 | 550550 | mecr |  |  | 9422 | 0.339 | 0.2782 | No |
| 34 | 431754 | acaa1 |  |  | 9613 | 0.326 | 0.2783 | No |
| 35 | 793834 | hadhab |  |  | 10077 | 0.298 | 0.2694 | No |
| 36 | 405845 | zgc:77118 |  |  | 10210 | 0.290 | 0.2706 | No |
| 37 | 393105 | hsd17b4 |  |  | 10332 | 0.283 | 0.2721 | No |
| 38 | 560000 | cpt1ab |  |  | 10472 | 0.274 | 0.2729 | No |
| 39 | 559656 | acsl3b |  |  | 11481 | 0.218 | 0.2452 | No |
| 40 | 449677 | cpt1b |  |  | 11572 | 0.212 | 0.2464 | No |
| 41 | 406325 | acaa2 |  |  | 11783 | 0.202 | 0.2435 | No |
| 42 | 100150288 | elovl6l |  |  | 12263 | 0.176 | 0.2317 | No |
| 43 | 406421 | acox3 |  |  | 12372 | 0.170 | 0.2315 | No |
| 44 | 322626 | hsd17b12b |  |  | 12386 | 0.170 | 0.2343 | No |
| 45 | 566085 | tecra |  |  | 12679 | 0.155 | 0.2280 | No |
| 46 | 566863 | acsl6 |  |  | 12701 | 0.154 | 0.2302 | No |
| 47 | 406725 | elovl1b |  |  | 13166 | 0.132 | 0.2181 | No |
| 48 | 327417 | hsd17b12a |  |  | 13341 | 0.124 | 0.2149 | No |
| 49 | 100126133 | ppt2 |  |  | 13345 | 0.123 | 0.2172 | No |
| 50 | 550361 | cpt1cb |  |  | 13535 | 0.114 | 0.2133 | No |
| 51 | 559001 | fasn |  |  | 15355 | 0.030 | 0.1567 | No |
| 52 | 394156 | acadl |  |  | 15452 | 0.024 | 0.1541 | No |
| 53 | 553326 | hacd1 |  |  | 16399 | -0.014 | 0.1246 | No |
| 54 | 334561 | acsl4b |  |  | 16648 | -0.022 | 0.1172 | No |
| 55 | 445175 | acsl1b |  |  | 16699 | -0.023 | 0.1161 | No |
| 56 | 767653 | elovl8a |  |  | 17124 | -0.037 | 0.1035 | No |
| 57 | 558088 | cpt1aa |  |  | 17283 | -0.044 | 0.0993 | No |
| 58 | 564649 | osxm |  |  | 17307 | -0.045 | 0.0994 | No |
| 59 | 555308 | acsl1a |  |  | 18027 | -0.077 | 0.0783 | No |
| 60 | 445121 | hadh |  |  | 18086 | -0.080 | 0.0780 | No |
| 61 | 368912 | echs1 |  |  | 18310 | -0.089 | 0.0726 | No |
| 62 | 317738 | elovl6 |  |  | 18840 | -0.116 | 0.0581 | No |
| 63 | 564111 | mcat |  |  | 18915 | -0.118 | 0.0580 | No |
| 64 | 567157 | acsl3a |  |  | 19451 | -0.142 | 0.0438 | No |
| 65 | 335159 | acsbg2 |  |  | 19541 | -0.145 | 0.0438 | No |
| 66 | 445290 | acat1 |  |  | 21467 | -0.239 | -0.0123 | No |
| 67 | 100332846 | acsl2 |  |  | 21878 | -0.261 | -0.0203 | No |
| 68 | 798606 | tecrl2a |  |  | 22001 | -0.267 | -0.0192 | No |
| 69 | 573723 | acadvl |  |  | 22257 | -0.282 | -0.0219 | No |
| 70 | 561303 | si:ch73-41h24.1 |  |  | 22757 | -0.311 | -0.0318 | No |
| 71 | 101884755 | LOC101884755 |  |  | 22823 | -0.315 | -0.0280 | No |
| 72 | 406283 | acadm |  |  | 23289 | -0.347 | -0.0361 | No |
| 73 | 326954 | tecrb |  |  | 23996 | -0.391 | -0.0510 | No |
| 74 | 450002 | tecrl2b |  |  | 24206 | -0.407 | -0.0500 | No |
| 75 | 555308\_1 | acsl1a |  |  | 24418 | -0.416 | -0.0489 | No |
| 76 | 100333227 | LOC100333227 |  |  | 25306 | -0.467 | -0.0680 | No |
| 77 | 64815 | hsd17b8 |  |  | 25500 | -0.485 | -0.0650 | No |
| 78 | 393425 | elovl5 |  |  | 26119 | -0.539 | -0.0744 | No |
| 79 | 393839 | scp2a |  |  | 26251 | -0.552 | -0.0682 | No |
| 80 | 393769 | elovl4a |  |  | 26365 | -0.563 | -0.0612 | No |
| 81 | 386661 | scd |  |  | 26590 | -0.587 | -0.0573 | No |
| 82 | 562292 | acsf3 |  |  | 27857 | -0.723 | -0.0836 | No |
| 83 | 317738\_1 | elovl6 |  |  | 28857 | -0.889 | -0.0984 | No |
| 84 | 559001\_1 | fasn |  |  | 29124 | -0.950 | -0.0890 | No |
| 85 | 110437777 | LOC110437777 |  |  | 29497 | -1.063 | -0.0809 | No |
| 86 | 393441\_1 | hacd3 |  |  | 29812 | -1.168 | -0.0689 | No |
| 87 | 334121 | hacd2 |  |  | 29838 | -1.181 | -0.0477 | No |
| 88 | 406648 | ppt1 |  |  | 29919 | -1.210 | -0.0276 | No |
| 89 | 560000\_1 | cpt1ab |  |  | 30321 | -1.399 | -0.0141 | No |
| 90 | 100005717\_1 | cpt2 |  |  | 30500 | -1.505 | 0.0085 | No |
| 91 | 334217 | elovl7a |  |  | 30920 | -1.885 | 0.0305 | No |
Table: GSEA details [plain text format]

  

Fig 2: FATTY\_ACID\_METABOLISM(DRE01212)      
 Blue-Pink O' Gram in the Space of the Analyzed GeneSet

  

Fig 3: FATTY\_ACID\_METABOLISM(DRE01212): Random ES distribution      
 Gene set null distribution of ES for **FATTY\_ACID\_METABOLISM(DRE01212)**
